# The RNA-Binding Proteins Promote Resistance of Microglial Cells to Hypoxia

**DOI:** 10.34172/apb.025.46054

**Published:** 2025-12-23

**Authors:** Mohammad Khosravi, Zohreh Ghotbeddin, Sorour Chinipardaz

**Affiliations:** ^1^Department of Pathobiology, Faculty of Veterinary Medicine, Shahid Chamran University of Ahvaz, Ahvaz, Iran; ^2^Department of Basic Sciences, Faculty of Veterinary Medicine, Shahid Chamran University of Ahvaz, Ahvaz, Iran; ^3^Faculty of Veterinary Medicine, Shahid Chamran University of Ahvaz, Ahvaz, Iran

**Keywords:** Microglia, RNA binding protein, Exosome, Hypoxia

## Abstract

**Introduction:**

Microglial cells play a crucial role in responding to brain hypoxia. This study aimed to evaluate the effect of RNA-binding proteins (RBPs) on the resistance of microglial cells to hypoxia.

**Methods:**

Newborn rats were subjected to hypoxia under four conditions: hypoxia (H), one week after hypoxia induction (H1), control (C), and control one week after hypoxia induction (C1). Microglial cells were isolated and cultured, and exosomes were extracted from brain samples of healthy newborn rats of C1 group. RBPs were extracted from the aforementioned groups and transferred into the microglial cells using exosomes from the C1 group. Cell viability, expression of specific RBP genes, and innate immune factors were evaluated in the studied groups. Additionally, exosomes containing RBPs were injected into the hypoxic rats to investigate behavioral changes in-vivo.

**Results:**

The treatment of microglial cells with C1 exosomes elevated the viability rate. The RBP-H proteins significantly elevated the expression of the CPE, HIF-1α, PDI, and VEGF-A genes. Improvements in anti-protease activity, along with decreases in lysozyme and myeloperoxidase activity, were observed in hypoxic microglial cells following treatment with RBP-H and exosomes containing RBP-H. In vivo evaluations revealed that the hypoxic group treated with exosome loading with RBP-H exhibited significant improvements in recognition and balance maintenance.

**Conclusion:**

The RBPs may be considered a promising option for further studies in the treatment of brain disorders resulting from hypoxia.

## Introduction

 Oxygen requirements and sensitivity to oxygen deficiency vary across tissues.^1,2^ The central nervous system (CNS) is especially susceptible to oxygen deprivation; neurons experience significant damage from hypoxia, leading to structural and functional changes that contribute to neurodegenerative disorders.^3,4^ Transient ischemic hypoxemia poses additional risks of brain injury.^5^ In response to hypoxia, cells activate a transcriptional program regulated by hypoxia-inducible factors (HIFs).^2^ Mammalian cells can alter protein expression under hypoxic conditions, triggering processes such as cell cycle arrest, necrosis, apoptosis, and enhanced survival responses.^6^ Gene expression patterns are significantly influenced by post-transcriptional processes. RNA-binding proteins (RBPs), which serve as primary regulators of gene expression, can respond to various stresses, including hypoxia. RBPs that modulate mRNA stability and translation in response to hypoxic conditions include the human R1 antigen, polypyrimidine tract-binding protein (PTB), and mRNAs encoding hypoxia-responsive proteins such as HIF-1 and vascular endothelial growth factor (VEGF). These factors enhance the expression of their respective genes following hypoxic exposure and play a crucial role in the development of hypoxic gene expression patterns.^7^

 Ischemic hypoxia can lead to secondary nerve damage following reoxygenation. This process involves the activation of microglia, which triggers the migration of peripheral macrophages, the release of pro-inflammatory cytokines. Some evidence suggests that inhibiting the inflammatory response may promote neuroprotection and could be utilized in the clinical treatment of ischemic brain injury.^8,9^ Activated microglia not only damage already compromised neurons but can also adversely affect healthy neurons. Most of our current understanding of inflammation, particularly the activation of microglia, following ischemic hypoxic injury in the developing brain, is derived from the Vannucci model. In this model, ischemic hypoxia is induced in newborn rats through low oxygen exposure and carotid artery occlusion.^10,11^

 Exosomes are nanoscale extracellular vesicles secreted by various cell types. They serve as carriers of several pathological and biologically active molecules, including DNA, RNA, and proteins, which significantly influence the biology of recipient cells. The use of exosomes as delivery vehicles for therapeutic agents addresses the limitations associated with polymers and liposomes in pharmacology due to their low immunogenicity, ability to distribute across all organs—including crossing the blood-brain barrier—and, in some cases, their affinity for specific tissues.^12-14^ Previous research has highlighted the potential application of exosomes, particularly microglial-derived exosomes, in the treatment of CNS degenerative diseases.^13,14^

 In the current study, RNA-binding proteins were isolated from newborn rats subjected to hypoxic conditions. The transfer of these proteins to microglial cells was accomplished using exosomes derived from normal brain cells. Following this transfer, it is anticipated that the resistance of microglial cells to hypoxia will correlate with the quantity and type of RBPs. In cases of hypoxia resistance, it is expected that cell tolerance to hypoxia will increase after the initial hypoxic stage. Furthermore, if tolerance is induced in microglial cells in response to hypoxia, the expression of RBP genes associated with hypoxia is expected to differ from that in cells that have not been treated with RBPs. By comparing the RBP gene expression in the acute hypoxic group (H) with those assessed one week after hypoxia (H1), this study aims to enhance our understanding of the relationship between hypoxia resistance and RBP proteins, as well as the potential therapeutic applications of RBPs. The most effective group of RBPs was selected for evaluation in an in-vivo experiment involving adult animals.

## Materials & Methods

###  Animals

 Rats were obtained from the Laboratory Animal Breeding Center at Ahvaz Jondi Shapur University in Ahvaz. Neonatal Wistar rats (postnatal days 10 to 12), each weighing approximately 20 grams, were maintained under standard conditions. The environment was clean and secure, providing appropriate bedding, food, water, and temperature regulation. The animals were kept at a temperature of 22 °C and a humidity level of 50%. Enrichment measures were implemented to reduce stress and enhance overall welfare. During the treatment phase, the animals were anesthetized using an intraperitoneal injection of a ketamine-xylazine mixture (25:5 mg/mL) at a dosage of 1 mL/kg body weight. Additionally, blood samples were collected concurrently with the administration of anesthesia. The animals were randomly divided into four groups, with five pups assigned to each group: hypoxia (H), one week after hypoxia induction (H1), control of hypoxia (C), and control of one week after hypoxia induction (C1).

###  Induction of Hypoxia

 The hypoxia induction chamber consisted of a glass enclosure equipped with adjustable gas inlet and outlet valves for connecting oxygen and nitrogen gas cylinders. The air inlet and outlet valves remained open for five minutes. After adjusting the gas mixture to 7% oxygen and 93% nitrogen in the hypoxia chamber and verifying the oxygen level with an oximeter, the valves were closed, and the rats were exposed to the gas mixture for 30 minutes. This protocol was repeated for five consecutive days. The temperature was maintained at 22 °C, and the relative humidity was kept between 40% and 50%.^15^

 Twenty-four hours after five days of hypoxia exposure, cardiac blood samples and brain organs were collected from rats in both the hypoxia and control groups. Blood and brain sampling were repeated one week later for both groups (H1 and C1) (see Supplementary file, Figure S1). Prior to collection, the rats were euthanized using a ketamine-xylazine injection. The chest area was cleaned for blood collection, which was performed using a sterile syringe. Blood was transferred to a Falcon tube containing 10% sterile EDTA and centrifuged for plasma isolation, which was subsequently stored at -70°C.

###  Preparation of Fe-RNA Conjugates

 RBP isolation was conducted using magnetic Fe-nanoparticles conjugated to RNA samples that had been extracted from brain tissues. RNA extraction was performed using the RNA extraction kit (Sinaclon, Iran). After obtaining brain samples from healthy rats, the samples were washed once with PBS, then crushed and transferred to a microtube, following the RNA extraction protocol. To assess the quality and purity of the RNA, horizontal electrophoresis on an agarose gel and optical density measurements at 260 nm and 280 nm were utilized. The RNA concentration was optimized by adjusting the absorbance at 260 nm on 1, equivalent to 40 μg/mL

 Fe-nanoparticles were synthesized as described by Khosravi et al. (2021) using n-octylamine as a surfactant.^16^ The preparation of Fe-RNA conjugates was performed by reacting Fe-nanoparticles with RNA through the 5´-phosphate group using an EDC-NHS linker.^17^ In addition to the non-covalent bonds formed between the phosphate or amino groups of RNA and the amino groups on the surface of Fe nanoparticles, the phosphate groups of RNA can also react via EDC-NHS on the surface of Fe nanoparticles to form a phosphoramidate bond. Specifically, 400 μL of the extracted RNA sample was added to 1 mL of filtered 10 mM MES buffer (pH 6; prepared by dissolving 20 mg of MES in 10 mL of distilled water). Then, 100 μL of EDC (4 mg/mL) was added and allowed to react for 30 minutes. Next, 100 μL of NHS (6 mg/mL) was added, and the reaction continued for an additional 30 minutes. Subsequently, 1 mL of Fe-nanoparticles (with an optical absorption at 600 nm of 0.312) was collected using a magnet, suspended in the MES buffer, and added to the reaction mixture, which was shaken for 2 hours. The Fe-nanoparticle-RNA mixture was collected using a magnet, washed three times, and then suspended in filtered MES buffer containing 0.1 M Tris buffer at pH 8.8. After two hours, the nanoparticles were collected again and suspended in 2 mL of sterile PBS containing 1% BSA, along with 5 μL of RNase inhibitor (Jena Bioscience, Germany) (Supplementary Figure 2). Prior to the addition of stabilizers, the conjugation of RNA to Fe-nanoparticles was confirmed by analysis using Fourier-transform infrared spectroscopy (FTIR), ultraviolet-visible (UV-Vis) spectroscopy at 260 nm (Nabi, Microdigital Co. Ltd, South Korea), and imaging via field emission scanning electron microscopy (FESEM).

###  Analysis Using Fourier-Transform Infrared Spectroscopy (FTIR)

 Fe nanoparticles, Fe nanoparticles activated by EDC.NHS without RNA, and Fe nanoparticles conjugated to RNA nanoparticles were dried in a 45°C incubator for 72 hours. Additionally, the RNA sample in phosphate-buffered saline (PBS) was prepared for FTIR analysis (Perkin-Elmer, 2400). FTIR was used to characterize the chemical groups of the nanoparticles and their composites. For this analysis, the potassium bromide (KBr) pellet method was employed, producing spectra with an absorption range of 400 to 4000 cm^-1^.

###  Extraction of RBPs

 Four microtubes were designated as follows: H (hypoxic rats), C (healthy rats of the same age as the hypoxia group), H1 (one week after hypoxia induction), and C1 (healthy rats of the same age as those one-week post-hypoxia induction). Subsequently, 1 gram of sonicated brain samples, prepared from a mixture of all samples in each group, was placed into each microtube. The mixed samples were then suspended in 1 mL of PBS. Additionally, 250 μL of Fe nanoparticles conjugated to RNA were added to the brain mixtures. The mixture was incubated on a shaker for one hour. Afterwards, the nanoparticles were washed five times with PBS. The washing steps continued until all free proteins were completely removed from the nanoparticles. This washing process involved adsorbing the magnetic nanoparticles using a strong magnet, followed by removal of the supernatant. The protein concentration in the resulting solution was determined by the Bradford method. A release buffer containing 0.1 M Tris at pH 8.3 was prepared and heated in a water bath to 55 °C. Then, 0.5 mL of the release buffer was added to the microtubes, which were placed in the water bath at 55 °C for 5 minutes. Subsequently, the microtubes were shaken for an additional 10 seconds, after which the nanoparticles were collected using magnets. The supernatant was retained as a solution containing RBP. After dialysis in sterilized PBS for 24 hours using dialysis tubing, the concentration of the isolated proteins was determined using the Bradford method, and the molecular weight of the RBPs was analyzed via SDS-PAGE.

###  Microglial Cultivation

 Isolation of microglial cells was performed following the method of Woolf et al. (2025)^18^, with modifications based on primary in-house protocol optimization. Euthanasia was induced in the animals by administering a high dose of ketamine-xylazine. The rats were then decapitated using sharp scissors. The scalp was incised along the midline, starting at the back of the head and extending toward the snout. After carefully extracting the brains from the newborn rats, they were placed in petri dishes containing 5 mL of cold DMEM without fetal bovine serum (FBS). Under an autopsy microscope, the meninges were carefully removed before isolating the cortex and hippocampus.

 Half of the brains of the C1 animals were placed in a microtube and frozen at -70°C. The other half was transferred to a petri dish containing 5 mL of DMEM medium without FBS, where the tissue was cut into small pieces using scissors. Trypsin was then added, and the mixture was incubated at room temperature for 15 minutes. Following this, 2 mL of FBS was added to neutralize the trypsin, and the contents of each plate were transferred to a 50 mL tube. The tube was filled with DMEM to achieve a final volume of 30 mL of culture medium. The tube was then centrifuged at 1000 RPM for 3 minutes to remove the remaining brain tissue. The supernatant was carefully removed for cell culture and transferred to a new Falcon tube, which was subsequently centrifuged at 3000 RPM for 5 minutes. The supernatant was discarded, and cells were suspended in 5 mL of DMEM medium in a Falcon tube. Before transferring the contents of the tube to the T-75 flasks, 1.5 mL of FBS was poured into the flasks and incubated at room temperature for one hour and allow it to dry at 37ºC, which aids to adherence of microglial cell to flasks. Following this, 15 mL of culture medium was added to each flask, and the flasks were placed in a CO2 cell culture incubator set to 5% CO2 at 37 °C. After 4 hours, a gentle tap was applied to the flask to dislodge any loose adherent cells. The culture medium was changed after washing the adherent cells twice with sterilized PBS. The adherent cells were observed daily to monitor their morphological changes and were used for subsequent experimental tests.

###  Exosome Extraction

 On the second day after culturing microglial cells of the C1 group, the supernatant medium was removed, and the flask was washed twice with PBS. Five mL of culture medium without FBS were added to the flask, which was then incubated in a CO2 incubator for 24 hours. The following day, the supernatant was collected and centrifuged at 4000 rpm for 15 minutes. The resulting liquid was then filtered through a 0.2-micron filter, and this filtered solution was used to extract the exosomes using an exosome extraction kit (Exocib, Iran).

###  Characterization of the Exosomes

####  Determination of Concentration and Molecular Weight of Proteins

 The Bradford method was conducted for determination of protein level following the usual method. Electrophoresis on the sodium dodecyl sulfate- polyacrylamide gel electrophoresis (SDS-PAGE) was conducted following the usual method for RNA-binding proteins (RBPs) and exosomes.

###  Exosome Imaging Using FESEM 

 Aluminum sheets measuring 2 cm by 2 cm with a smooth surface were prepared. Subsequently, 50 µL of exosomes were spread onto the aluminum surface using a clean slide, and the samples were allowed to dry for one hour. Each sheet was immersed in 2% glutaraldehyde for 1 hour. Incremental grades of acetone were prepared in concentrations of 30%, 40%, 50%, 60%, 70%, 80%, 90%, and 100%, using acetone and PBS. Each aluminum sheet was incubated in these acetone solutions for 10 minutes at each concentration. Imaging was performed using an FESEM microscope (MIRA 2, Czech).

###  Tetraspanins Analysis of Exosome

 The isolated exosome was provided at concentration of 4 μg in 49 μL PEB (PBS + 5 mM EDTA + 0.5 % BSA. The APC-conjugated CD63, PE-conjugated CD9, and FITC-conjugated CD81 monoclonal antibodies (mAbs) purchased from Miltenyi Biotec (Germany) were added to the solution. The sample was incubated for 1 h at 4 °C under light-protected condition and diluted with 450 μL PEB for flow cytometry analysis. Fluorescence-labelled isotype-matched mAbs were used as controls.

###  Conjugation of isolated RBPs to FITC

 One microliter of FITC (5 mg/mL) was added to a microtube containing 1 mL of 0.1 M MES buffer at pH 6.8. Next, 100 μL of EDC solution (4 mg/mL) was added to the mixture, which was then placed on a shaker. After 30 minutes, 100 μL of NHS solution (6 mg/mL) was added, and the mixture was shaken again. Subsequently, a mixture of four groups of isolated RBP was prepared by combining 25 μL (10 μg) of each sample into the reaction and allowing it to shake for one hour. Finally, blocking was performed using a Tris solution at pH 8.8 to achieve a final concentration of 0.1 M. The prepared sample was dialyzed in PBS for 48 hours at 4 °C and stored at 4 °C.

###  Loading of RBPs into the Exosomes 

 To load the exosomes with FITC-labeled RBP, the suspension method was employed using a carbonate buffer. A solution containing 50 μg/mL of FITC-labeled RBP and 100 μg/mL of exosomes from the C1 group in PBS was prepared. Then, 0.5 mL of sterilized and filtered bicarbonate buffer (pH 9.6) was mixed with 250 μL of exosomes and 250 μL of FITC or FITC-conjugated RBP. The mixture was shaken for one hour at room temperature. Subsequently, the exosomes precipitation kit was used to extract exosome and remove any unincorporated dyes and proteins. The supernatant was discarded, and the precipitated element, which contained the exosomes and incorporated proteins, was collected for fluorometric analysis. In addition, the cultured microglial cells were incubated with the treated exosome (20 µg/5.000 cells) for 12 hours. The unconjugated proteins, untreated exosomes and cells were used as controls. The prepared samples and treated cells were analyzed using a fluorometric method with an excitation wavelength of 520 nm.

###  Effects of Exosomes Containing RBPs on Microglia Activity

 After confirming the RBP loading into the exosome C1, these RBPs were utilized to investigate their effects on the differentiation and cytokine profiles of microglia, as well as to assess the impact of RBP uptalking on resistance to hypoxic conditions. The protein quantification was performed on prepared samples using the Bradford method to achieve the required concentration. The experimental groups included exosomes from the C1 group containing RBPs from cerebral of hypoxic group (ExoC1-RBP-H), or RBPs from non-hypoxic cerebral cells (ExoC1-RBP-C), or RBPs from the hypoxic group one week after hypoxia (ExoC1-RBP-H1), and RBP from the cerebral of control group with the same age as one week after hypoxia induction (ExoC1-RBP-C1). Additionally, a control group was treated with a culture medium containing exosomes or RBPs alone. It is important to note that the negative and positive controls for this experiment were microglial cells under hypoxic and non-hypoxic conditions, respectively, which were not influenced by any treatment. Adjustment of cell quantity was obtained by addition 1 mL of trypsin to the cultured microglia flasks to detach the microglia from the T-75 flasks. Subsequently, the cells were distributed into four 96-well plates, with approximately 5,000 cells in each well. After 48 hours, when the cells had elongated, the aforementioned treatments at a concentration of 20 mg/mL in DMEM medium and a volume of 100 μL were added to each well. Each treatment was conducted in five replicates, while the control groups without treatment were repeated in ten replicates. After 24 hours, all cells, except for half of the wells in the control group, were treated with a concentration of 100 μg/mL cobalt chloride to induce hypoxia. One plate containing two complete sets of treated and control cells was treated by trypsin after 24 hours and removed for immunology tests and Real-Time PCR analysis. The remaining two plates were placed in a CO_2_ incubator at 5% CO_2_ and 37 °C for one week, and two weeks. The culture medium of each well was changed at 72-hour intervals. The cells detached from the wells, along with the supernatant, were reintroduced into the well during the second week of the experiment by centrifuging the harvested medium at 2000 rpm for 4 minutes.

 The MTT test was conducted at the designated time. MTT powder was defined to a concentration of 5 mg/mL and dissolved in 10 mL of DMEM medium. Subsequently, 100 μL of the MTT solution was added to each well. The plate was then incubated for 4 hours in a CO2 incubator at 37 °C. After this incubation period, the supernatant medium was removed, and 100 μL of DMSO was added to each well. The plate was returned to the incubator for an additional 30 min in the dark under the same conditions, after which the optical density was measured at a wavelength of 600 nm.

###  Microglia Gene Expression Analysis

 To prepare the cells for Real-Time PCR and innate immunity tests, the culture medium was removed from the wells after 24 hours. Subsequently, 30 μL of trypsin was added to each well and incubated for 5 minutes. After this incubation period, 200 µL of DMEM medium was added to the wells to neutralize the trypsin. The cells were then collected and transferred into a microtube, followed by centrifugation at 2000 RPM for 5 minutes. The supernatant was discarded. The cells were then placed in a -20°C freezer for 30 minutes before being transferred to a -70°C freezer. A separate batch of cells was suspended in 100 μL of PBS and frozen at -70°C for immunology tests. According to the described method, RNA was extracted using the Sinaclon RNA extraction kit (Sinaclon, Iran). The genomic DNA was first removed using DNase treatment (Sinaclon, Iran); following this step, complementary DNA (cDNA), was synthesized from the extracted RNA according to the specified kit protocol.

 The expression levels of the HIF-1α, cytoplasmic polyadenylation element binding protein (CPEB), protein disulphide isomerize (PDI), heat shock protein-70 (HSP70), and VEGFa genes were investigated in comparison to the beta-actin gene. This analysis was conducted 24 hours after treating microglial cells with hypoxic conditions. The components used in each reaction of Real Time-PCR were in a total volume of 20 μL, which included 10 μL of Mastermix (Amplicone, Germany), 1 μL of each primer (Table 1), 2 μL of each of the extracted cDNA, and 6 μL of distilled water. The reactions were prepared on ice, and the samples were immediately placed in a thermocycler (STEP ONE PLUS Real-Time PCR). The thermal cycling conditions included: 94°C for 5 minutes (1 cycle), 95°C for 30 seconds (35 cycles), 60°C for 30 seconds (35 cycles), 72°C for 30 seconds (35 cycles), and a final hold at 8°C for 8 minutes (1 cycle).

**Table 1:** Sequences of the primers used in the Real-Time PCR analysis.

**Primer name**	**Sequences**	**Length of Product**	**References**
B-actin-Rat	Forward 5'-TCCTTCCTGGGTATGGAATC-3 Reverse 5'-GCACTGTGTTGGCATAGAGG-3	103	Zhu et al., 2009 ^19^
HIF-1α -Rat	Forward 5-TGGTGCTAACAGATGATGGTG-3 Reverse 5-CATGGTCACATGGATGGGTA-3	123	Dong et al., 2022 ^20^
CPEB4-Rat	Forward 5'-ACAGTGACTTTGTGATGGATGG-3 Reverse 5'-TTATCATCGCAAGCTCCACA-3	105	Tsai et al., 2014 ^21^
VEGF-Rat	Forward 5′-AGGCGAGGCAGCTTGAGTTA-3′ Reverse 5′-CTGTCGACGGTGACGATGGT-3′	166	Zhang et al., 2014 ^22^
PDI-Rat	Forward 5'- ACGGTGAGCGGACACTAGAT-3' Reverse 5'- GAGCTGGCCACACTCACATCAT-3'	91	M_032912254
HSP70-Rat	Forward 5'-GTTCCAGAGGCTGTTCAAGC-3' Reverse 5'-TCTTGCTCTGGACACATTGC-3	172	Ekici et al., 2024 ^23^

###  Evaluation of Innate Immune Factors

####  Evaluation of Lysozyme Activity 

 In this turbidimetry evaluation, a suspension was prepared with a concentration equal to 2 mg *Micrococcus lysodeikticus* into 10 mL acetate buffer (0.02 M, pH 5.5). Subsequently, 20 μL of serum samples or lysate cells and 80 μL of the prepared bacterial suspension were added to the wells of a 96-well microplate.^24^ The OD was defined at a wavelength of 600 nm after 5 minutes. The decreased OD equal to 0.001 per minute was taken as one unit of lysozyme activity.

###  Myeloperoxidase Activity Evaluation 

 The oxidation of 3,3´,5,5´-tetramethylbenzidine (TMB) by Myeloperoxidase(MPO) was used for determination of the serum MPO activity. Based on the modified methods of Malle et al. (2007),^25^ the serum samples or cell lysates 10 μL was added to 90 μL of Hanks balanced salt solution without Ca^2+^ or Mg^2+^ in 96-wells microplates. After that, 35 μL of 20 mM TMB and 5 mM H_2_O_2_ were added to each well. The reaction was stopped by addition of 35 μL of 4 M sulphuric acid after 5 minutes. The MPO activity was determined using a spectrophotometer at 450 nm wavelength and results were expressed as OD.

###  Antiprotease Activity Test

 Initially, 20 μL of 5% trypsin was added to 100 μL of a 50 mM Tris-HCl solution (pH 8.2) in a microplate well. Subsequently, 10 μL of serum or cell lysate was incorporated into this mixture. The control samples included all components except for the serum or cell lysate samples. The resulting mixture was incubated for one hour at room temperature. Following the incubation, 3-N-alpha-benzoyl-DL-arginine p-nitroanilide (BAPNA) substrate (0.1 mM) and calcium chloride (20 mM) were added to the Tris-HCl solution. A total of 20 μL of this solution was then added to all samples, and O.D was detected at a wavelength of 405 nm (T1). The samples were incubated at room temperature for an additional 30 minutes and the O.D at 405 nm was detected again (T2). The difference of T1 and T2 absorbances were calculated and the level of antitrypsin activity was accounted by determining the difference in absorbance between each sample and the control samples, divided by the absorbance of the control samples.^26^

###  In-Vivo Evaluation of RBP Effects on Resistance to Hypoxia 

 After optimizing the dosage of RBPs, the main experiment was conducted with sixteen adult female rats, each weighing approximately 200 ± 20 grams. The rats were randomly divided into four groups (n = 4). Three groups were exposed to hypoxia, with an oxygen concentration of 7% and 93% nitrogen, for 90 minutes. Due to the maturation stage of the rats, the protocol for inducing hypoxia differed from that used for immature rats. Exosomes loaded with RBP extracted from hypoxic conditions (ExoC1-RBP-H) and control conditions (ExoC1-RBP-C) were injected into the tail vein of the two groups of hypoxic rats at a volume of 100 μL. Behavioral tests were conducted on the animals 24 hours and one week after the treatments. An additional hypoxia control group and a control group without hypoxia that did not receive any treatment were included in the experiments (Supplementary Figure 3).

###  Novel Object Recognition Test

 This evaluation is designed to assess memory in rodents without reliance on external motivation or incentives. The test was conducted in a quiet, uniformly lit play chamber, which consists of three phases. In the first phase, called the familiarity phase, the animal explores the empty chamber for five minutes. Afterward, it returns to its cage. The second phase introduces two identical objects placed 10 cm from the walls in opposite corners. This arrangement encourages exploration without pushing the animal to search too hard, as the items are oriented away from each other. After that, the rat was returned to its cage again. In the third phase, one of these identical objects is replaced with a distinctly different item, and the rat was reintroduced to the chamber. Novel object recognition is evaluated based on the time the animal spends exploring the new versus the familiar object. Test results are analyzed by looking at the duration of exploration during training and testing phases, including a differentiation index that reflects the difference in time spent with the new and old objects.^27^

###  Rotarod Test

 This device is utilized to evaluate the effects of different drugs and substances on motor coordination. In the rotarod test, the rats were placed on the axis of the device, which then began to rotate from 5 RPM to 45 RPM in 300 seconds, and the duration for maintaining balance was noted. Initially, every animal received two chances to adjust to the device, then the animal was positioned on the rotating rod three times at intervals of 2 minutes, and the average of these durations computed. It is important to mention that the maximum time recorded for the rat’s presence on the rod was 300 seconds.^28^

###  Wire Hanging Test

 The wire hanging test evaluates various elements of locomotor capability, such as grip strength, endurance, and body coordination. It is commonly utilized in rodents that have neurological issues and/or muscle weakness. Animals are positioned on a wire suspended 50 to 60 cm above the ground for a maximum duration of 2 minutes, requiring them to hold their bodies up with their limbs. The duration that animals remain on the wire (latency prior to falling), indicating muscle strength, is documented. When suspended, animals may utilize their forelimbs or all four limbs to grasp the wire.^29^

###  Data Analysis

 The results of the tests were analyzed using SPSS software (version 2022) and one-way ANOVA and Tukey’s statistical tests. Plotting of the results was performed using GraphPad Prism 8 software. The gene expression data were evaluated using the StepOne software with the ΔΔCT comparative method. The results of Real-Time PCR were calculated based on the general formula of ΔΔCT as [ΔΔCT = (Ct target – Ct βactin) sample – (Ct target – Ct βactin)] and fold change expression were reported by 2^-ΔΔCT^. The *P* < 0.05 value was considered statistically significant. Data are presented as mean with standard deviations (SD).

## Results

###  Induction of Hypoxia in Newborn Rats

 After at least one minute of placing the newborn rats in the hypoxia induction box, seizures occurred. These movements started with small movements of the hands, feet, and face, and ended with tonic-clonic movements, which then encompassed the entire limb and the rats had jumping movements. The induction of hypoxia was confirmed by the occurrence of these behaviors. According to statistical analysis (*P* = 0.0001; F(9, 30) = 14; DF = 9), the final weight of the hypoxic rats 21 gr showed a significant difference (*P* ≤ 0.001) in comparison to the control group which was equal to 30 gr.

###  Analysis of the Prepared Fe-RNA Nanoparticles

**Figure 1:**
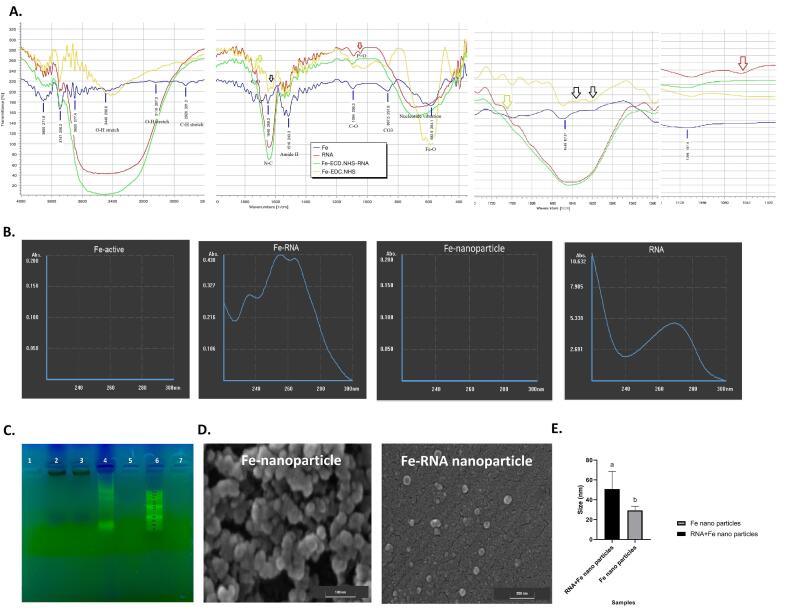
Preparation of Fe-RNA conjugates and extraction of the RBP using Fe-RNA conjugates. (A) Fourier-transform infrared (FTIR) spectroscopy analysis of Fe nanoparticles, Fe nanoparticles treated with EDC-NHS without RNA, Fe-RNA conjugates, and RNA. The red and green arrows indicate the phosphate groups on RNA and the C = O stretch of nucleotides, respectively. The black arrows highlight the main changes in amin groups on Fe after reaction with RNA. (B) UV-visible spectroscopy at 260 nm of Fe nanoparticles, activated Fe nanoparticles with EDC.NHS without RNA, RNA, and Fe-RNA conjugates confirming successful RNA conjugation. (C) The gel electrophoresis of the mentioned reagents. 1, 5 and 7 negative control wells, 2: Fe-RNA conjugate; 3: activated Fe-EDC.NHS without RNA, 4: extracted RNA and 5: 50 bp ladder (D) Field emission scanning electron microscopy (FESEM) images of Fe nanoparticles, Fe-RNA nanoparticles, and (E) comparison of their sizes

 Electron microscopy and FTIR spectroscopy were performed on Fe nanoparticles conjugated to RNA extracted from rat brains to confirm successful RNA binding to the Fe nanoparticles. In interaction of Fe nanoparticles and RNA, the amide I peak of Fe disappeared after reaction with RNA, while the amide II peak intensified. The *P* = O groups of RNA at 1042 cm^-1^ and carbonyl groups at 1690–1720 cm^-1^ disappeared following reaction of RNA with Fe nanoparticles, indicating successful conjugation and involvement of phosphate and carbonyl groups in the interaction between RNA and Fe nanoparticles. FTIR results of Fe interaction with EDC.NHS without RNA, revealed changes in Fe nanoparticle peaks at 1683 and 1645, 1623, 1507-1519 cm^-1^, corresponding to amide bands of Fe-nanoparticles, attributed to EDC-NHS absorption by the iron nanoparticles. The C–N peak at 1516, 1649 cm^-1^ observed in Fe and Fe-EDC.NHS nanoparticles likely arise from the use of n-octylamine during iron nanoparticle synthesis and the presence of surface NH₂ groups. Additionally, analysis of the 3000–3800 cm^-1^ region revealed differences in hydrophobicity among Fe, Fe-EDC-NHS, and Fe-RNA conjugates; the conjugated product exhibited increased hydrophobicity compared to the initial nanoparticle backbone (Figure 1A). Furthermore, UV-visible spectroscopy showed a characteristic peak at 260 nm for the Fe-RNA conjugates, confirming successful RNA conjugation; this peak was absent in both Fe and Fe-EDC.NHS nanoparticles alone (Figure 1B). Additionally, the agarose gel electrophoresis revealed a faint smear appearing in the lane containing the Fe-RNA conjugates (Figure 1C). Statistical analysis (*P* = 0.0145; F = 18.41; DF = 9; t = 2.591) revealed that the size of Fe nanoparticles conjugated to RNA was significantly larger than that of unconjugated Fe nanoparticles (Figures 1D and 1E).

###  Culture and Morphological Changes of Microglia

**Figure 2:**
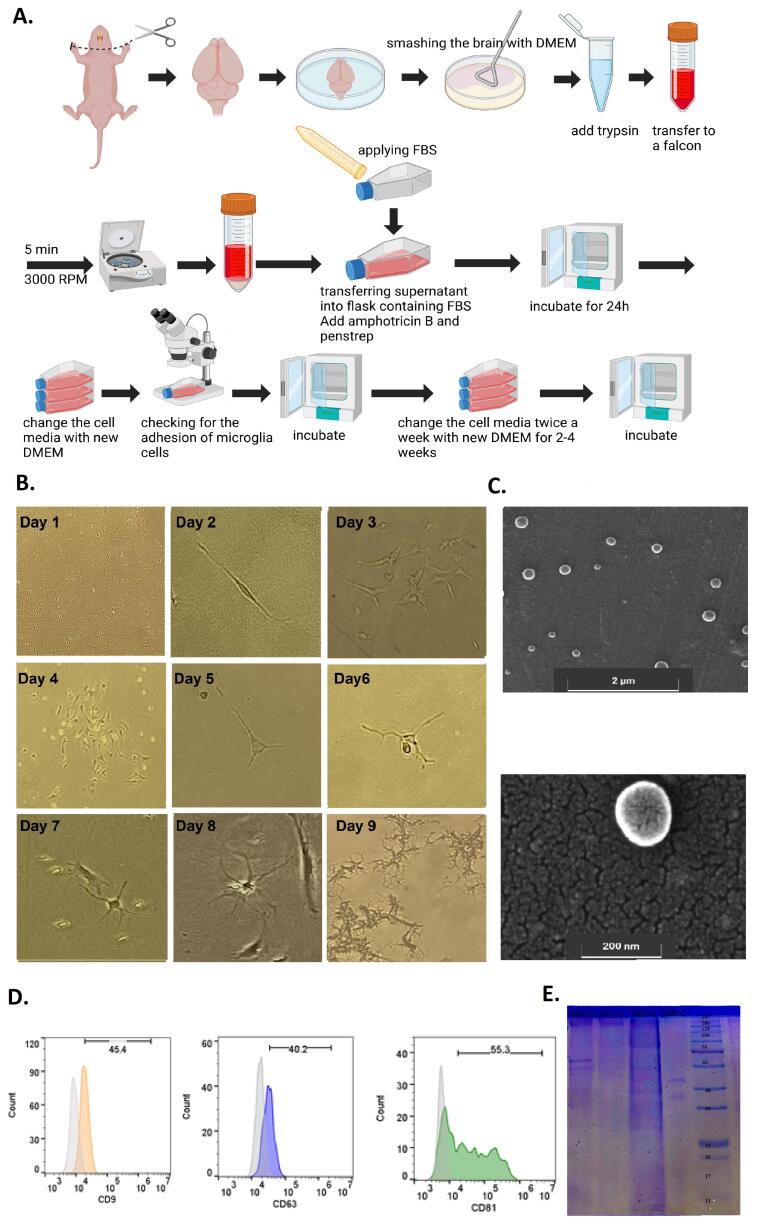
Extraction and characterization of microglial exosomes from rats in hypoxic and control groups. (A) Schematic diagram illustrating the process of microglial cell extraction. (B) Morphological changes observed in rat brain-derived microglial cells during evaluation. (C) FESEM images of exosomes extracted from microglial cells of newborn rats. (D) Expression of tetraspanin markers in exosomes extracted from newborn rats. (E) The protein band profiles of RBP from SDS-PAGE and quantification of extracted RBPs from the brains of newborn rats in four groups: hypoxia induction (H), one week after hypoxia induction (H1), control (C), and control one week after hypoxia induction (C1)

 The culture of microglial cells of C1 group was successfully established, and to accurately observe the changes in cell morphology during the growth stages, microglial culture flasks were examined daily using an inverted light microscope to document their growth and differentiation. Observations indicated that the cells exhibited an undifferentiated, rounded appearance on the first day. From the second to the fourth day, the cells elongated and assumed a spindle shape. During the fifth and sixth days, the cells developed a branched morphology with a central cell body. From the seventh to the ninth day, most of the cells displayed root-like appendages with distinct branches (Figure 2A and 2B).

###  Characterization of the Microglial Exosome

 The exosome was characterized using FESEM and Flow cytometry assays. Additionally, the protein profiles of exosomes were evaluated through SDS-PAGE. The extracted exosomes had 139.276 nm diameter (Figure 2C). The purified exosome showed expression of tetraspanins including CD9, CD63 and CD81 (Figure 2D).

###  Identification of RBPs

 A significant difference was found in the protein value of the RBP isolated from the brains of newborn rats one week after hypoxia induction compared to other groups. To determine the protein weights extracted from rat brains, SDS-PAGE was employed. Due to the limited quantity of RNA-binding proteins, no distinct bands were visible in this test. However, upon sample concentration by freeze drying, bands with varying molecular weights were observed in the studied groups (Figure 2E).

###  Evaluation of Exosome Loading with RBPs

**Figure 3:**
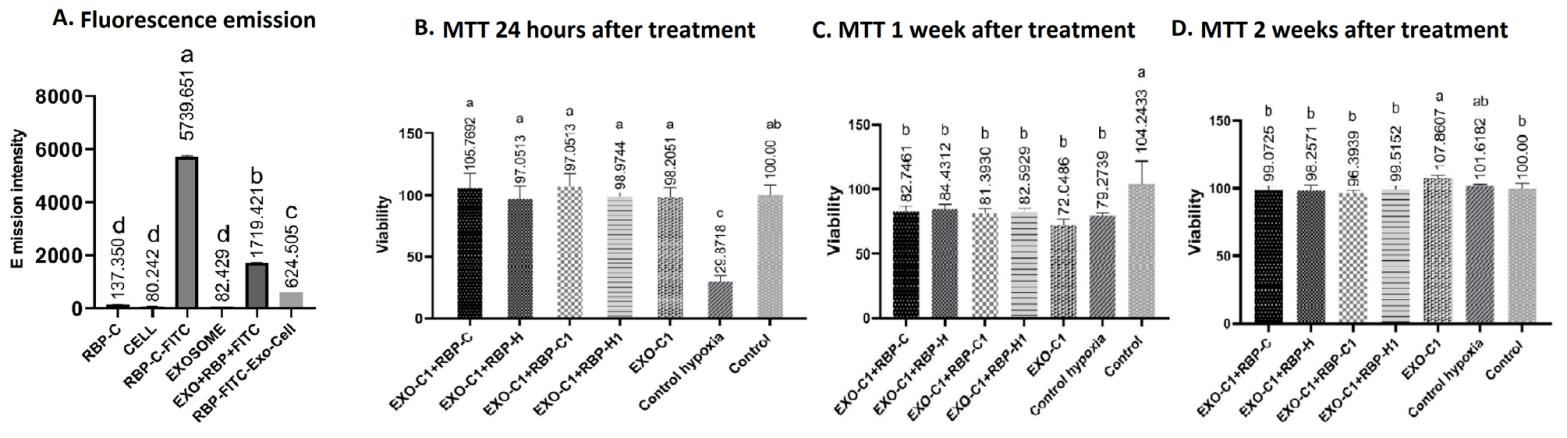
Loading of RNA-binding proteins (RBPs) into exosomes and their effects on viability of the microglial cells. (A) The elevated fluorescent peaks of the loaded exosomes with FITC-labeled RBPs, along with the significant differences compared to control samples including: RBPs, microglia, and exosomes samples confirm the successful incorporation of these proteins into the exosomes and their subsequent uptake by the microglial cells. Evaluation of microglial cell viability following treatment with microglial exosomes containing RBP from control rat (C), after induction of hypoxia (H), one week after hypoxia induction (H1), and control of one week after hypoxia induction (C1). The images of B-D illustrate cell metabolic activity at (B) 1, (C) 7, and (D) 14-days post-treatment, respectively. A significant resistance of microglial cell to hypoxia was observed 24 hours after treatment with exosomes containing RBP from the different groups; However, a significant decrease in cell activity was noted one week after treatments. Notably, treatment with control exosomes resulted in increased microglial cell activity two weeks after treatment

 The successful labeling of proteins with FITC and their incorporation into exosomes was confirmed by fluorometry analysis. Subsequently, microglial cells were treated with exosomes loaded with FITC-RBP. The treated cells were washed with PBS three times, and trypsinization was performed to assess the amount of fluorescent emission in comparison to untreated control cells. The results presented in Figure 3A indicate the success of this prosses.

###  Effect of Exosomes Containing RBPs on Microglial Metabolic Activity

 The effect of treatment with exosomes containing RBP on the metabolic activity of microglial cells was evaluated over a period of two weeks. Following 24 hours after hypoxia induction and treatment with exosomes C1 containing RBPs, significant differences were observed in metabolic activity of the treated cells (*P* < 0.0001) by ExoC1-RBP-H, ExoC1-RBP-H1, ExoC1-RBP-C, ExoC1-RBP-C1, and the treatment group with ExoC1 when compared to the hypoxic control cells. Notably, the treated cells did not show a significant difference in metabolic activity compared to the control cells that were not subjected to hypoxia (*P* = 0.0001; F(6, 21) = 39.46; DF = 6). One week after the induction of hypoxia in microglial cells, all groups—including ExoC1-RBP-H (*P* = 0.0190), ExoC1-RBP-H1 (*P* = 0.0088), ExoC1-RBP-C (*P* = 0.0094), ExoC1-RBP-C1 (*P* = 0.0053), ExoC (*P* = 0.0001), and hypoxia control group (*P* = 0.0022) exhibited a significant decrease in activity compared to the control group (*P* = 0.0004; F(6, 21) = 6.817; DF = 6). The absence of significant differences among the various treated groups is one of the key findings of this evaluation. At two weeks post-hypoxia induction, cells treated with ExoC1-RBP-C1 demonstrated a significant difference in metabolic activity compared to other groups (*P* = 0.0006; F(6, 21) = 6.585; DF = 6)., including the ExoC-RBP-H (*P* = 0.0040), ExoC1-RBP-H1 (*P* = 0.0073), ExoC1-RBP-C (*P* = 0.0045), and untreated control group (*P* = 0.0124) (Figure 3B, 3C and 3D).

###  Effect of Exosomes Containing RBPs on Microglia Gene Expression

**Figure 4:**
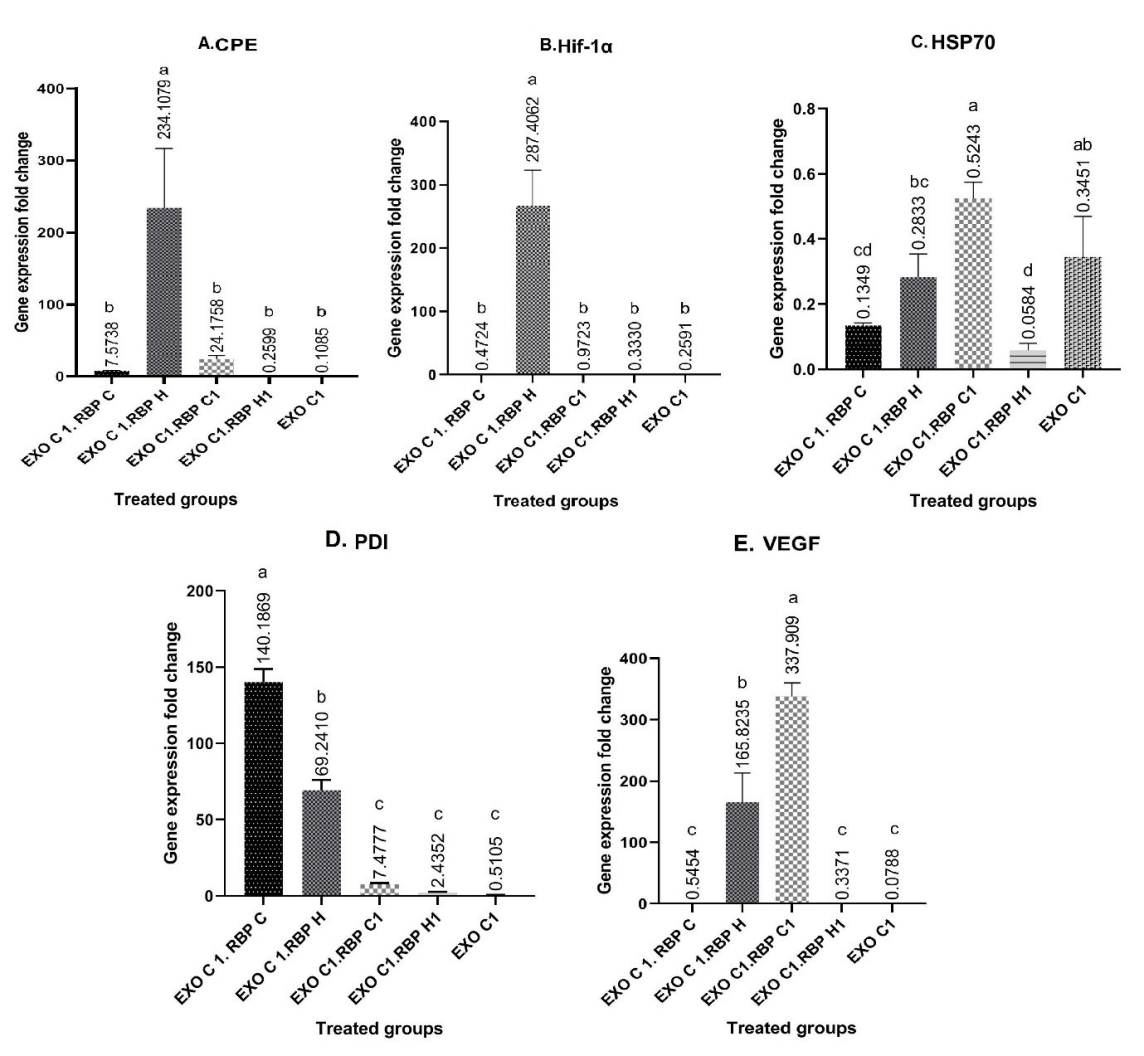
Expression changes of CPE, HIF-1α, HSP-70, PDI, and VEGF-A genes in hypoxic microglia cells following treatment with RNA binding proteins. The cells were treated by control exosome (ExoC1), ExoC1 containing RBP control (ExoC1-RBP-C), hypoxia (ExoC1-RBP-H), one week after hypoxia induction (ExoC1-RBP-H1) and control of one week after hypoxia induction (ExoC1-RBP-C1). The expression of genes has been analyzed in comparison with untreated hypoxic microglia cells. A statistically significant difference was shown compared to the other groups with different lowercase letters

 In order to investigate the effects of RNA-binding proteins extracted from the brains of the evaluated groups, the Real-Time PCR was employed to analyze gene expression regulation in hypoxic cells (Figure 4).

 The reduction of HSP70 expression in all treatment groups compared to the hypoxia control cells is one of the key findings of this evaluation (*P* = 0.0001; F(6, 10) = 21.21; DF = 4). Treatment of the hypoxic microglial cells with the C1 exosomes resulted in a 10.34-fold increase in the expression of the HSP70 gene. It is important to note that the expression levels of the CPE (*P* = 0.0001; F(4, 10) = 22.56; DF = 4), HIF-1α (*P* = 0.0001; F(4, 10) = 68.59; DF = 4)., and VEGF-A (*P* = 0.0001; F(6, 10) = 147.7; DF = 4). genes were reduced compared to the hypoxia control group that did not receive any treatment.

 The exosome containing the control RNA-binding protein (ExoC1-RBP-C) resulted in a 140.18-fold and a 7.57-fold increase in the expression of the PDI (*P* = 0.0001; F(6, 10) = 453.7; DF = 4) and CPE genes, respectively, compared to the hypoxia control. In contrast, HSP-70 gene expression decreased by 0.13 compared to the control. The expression levels of PDI and HSP-70 were significantly different from those of the control exosome.

 The ExoC-RBP-H significantly increased the expression levels of the CPE, HIF-1α, PDI, and VEGF-A genes by 234.1%, 287.4%, 69.2%, and 165.8%, respectively, when compared to hypoxic control cells. Also, ExoC1-RBP-H1 resulted in a significant decrease in HSP-70 expression (*P* = 0.0032) compared to their carrier exosome. The ExoC-RBP-C1 proteins significantly increased VEGF-A expression by 337.2% compared to hypoxic control cells (*P* < 0.0001), and there was a significant difference (*P*< 0.0001) when compared to the exosomes carrying them.

###  Indicators of the Innate Immune System

####  Antiprotease Activity

**Figure 5:**
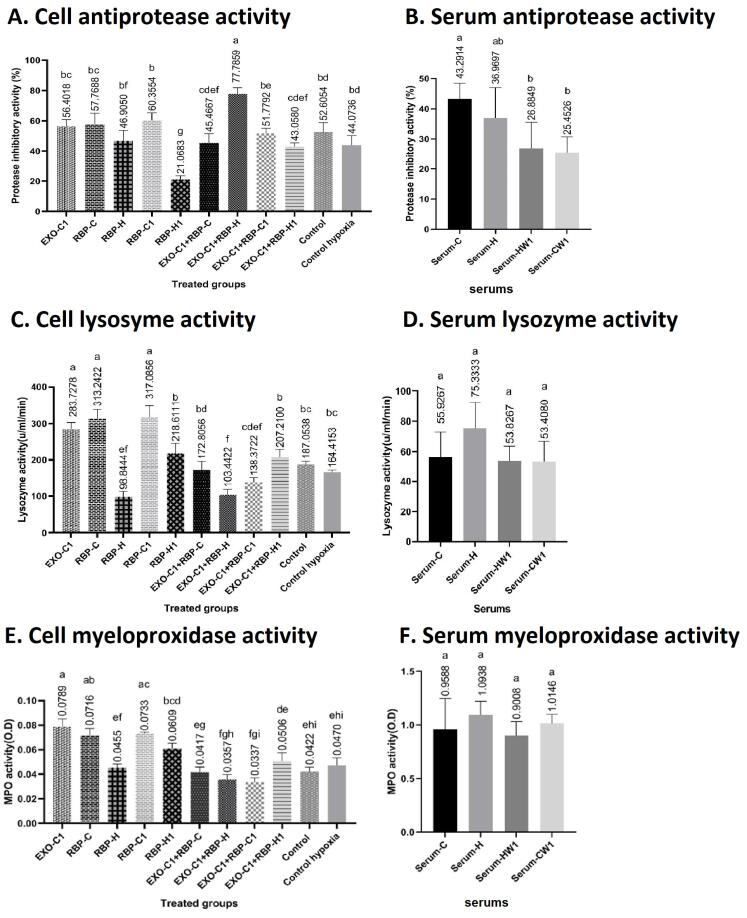
The innate immune responses of the hypoxic microglial cells following treatment with exosome containing RNA-Binding Proteins (RBP). (A and B) Antiprotease activity; (C and D) Lysozyme activity; (E and F) Myeloperoxidase activity in hypoxic and control rats, as well as microglial cells treated with exosome of control group (Exo-C1), RBP, and Exo-C1 containing RBP of the hypoxia group (ExoC1-RBP-H), RBP of a group one week after hypoxia induction (ExoC1-RBP-H1), and RBP of the control groups (ExoC1-RBP-C and ExoC1-RBP-C1). Significant differences compared to the other groups are indicated by different lowercase letters

 The antiprotease activity in the serum of hypoxic rats were not significantly different from that of the control groups. However, a significant decrease in antiprotease activity was observed with age (*P* = 0.0052; F(3, 16) = 6.258; DF = 3). The antiprotease activity of cells treated with exosomes containing RBP of hypoxic rat was significantly increased compared to other treatments. Additionally, the cells treated with ExoC1-RBP-H1 exhibited a significant decrease in antiprotease activity compared to the other groups (F(9, 20) = 23.96; DF = 9) (Figure 5A and 5B).

###  Lysozyme Activity

 Serum lysozyme activity in hypoxic rats did not differ significantly from that in the control groups (*P* = 0.0854; F(3, 16) = 2.634; DF = 3). A notable increase in lysozyme activity was observed in cells treated with RBP-C1, as well as in the RBP-C and exosome C1 groups, compared to the other groups. Conversely, a significant decrease in lysozyme activity was noted in microglial cells following treatment with RBP-H (*P* = 0.0001; F(9, 20) = 43.15; DF = 9) (Figure 5C and 5D).

###  Myeloperoxidase Activity

 The serum myeloperoxidase activity in hypoxic rats were not significantly different from that in the control groups(*P* = 0.383; F(3, 16) = 1.086; DF = 3). However, there was a significant increase in myeloperoxidase activity in cells treated with exosome, RBP-C, RBP-C1, and RBP-H1, which was significantly higher than the myeloperoxidase activity in the control cells (*P* = 0.0001; F(9, 20) = 39.34; DF = 9) (Figure 5E and 5F).

###  Behavioral Results

####  Recognition Test

**Figure 6:**
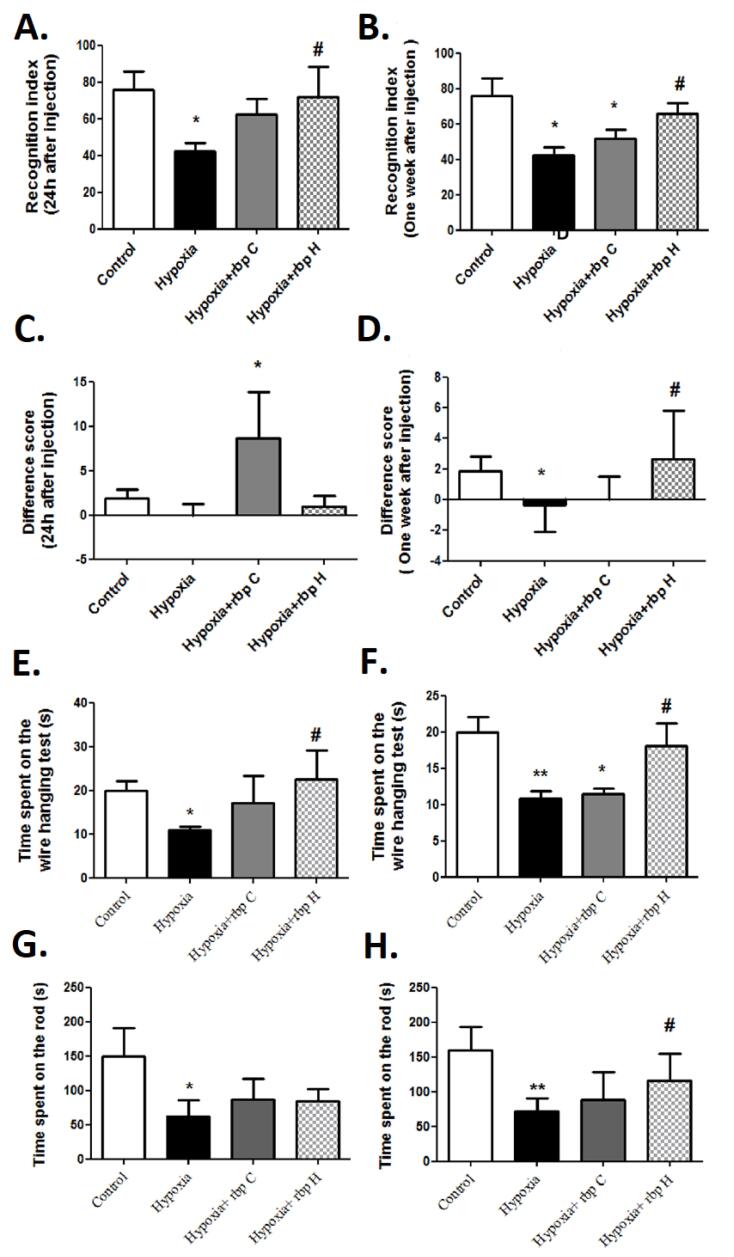
The effects of exosome containing RNA binding Proteins (RBP) injection on behavioral status of the hypoxic rats. (A and B) Recognition index; (C and D) Difference score; (E and F) time spent maintaining balance in the Rotarod test; (G and H) Wire hanging test. An asterisk (*) indicates a significant difference at the level of *P* < 0.05 compared to the control group. The hash symbol (#) denotes a difference between the hypoxia groups treated with RBP and the hypoxia group (*P* < 0.05). The data are presented as mean ± standard deviation (n = 4)

 The recognition index in the hypoxia groups significantly increased 24 hours after the injection of ExoC1-RBP-H in comparison to the untreated hypoxia group (*P* < 0.05) (*P* = 0.0138; F(3, 13) = 5.525; DF = 3). The percentage of time spent close to the new object compared to the total time spent investigating both objects was less in the hypoxia group than in the control group, suggesting that the hypoxia group dedicated less time near the new object compared to the control group. The hypoxic rat treated with ExoC1-RBP-H spent a longer duration investigating the new object than the hypoxia group, and the cognitive index in these treated rats showed a significant increase compared to the hypoxia group (*P* < 0.05) (Figure 6A).

 The recognition index in the hypoxia and hypoxia group treated with ExoC1-RBP-C was significantly (*P* < 0.05) decreased compared to the control group, one week after RBP injection. In particular, the hypoxia and hypoxia groups given ExoC1-RBP-C exhibited less time near the novel object compared to the control group. Conversely, the hypoxia group receiving ExoC1-RBP-H spent a greater duration near the novel object than the hypoxia group, leading to a notable rise in the cognitive index for this group in comparison to the hypoxia group (*P* < 0.05) (*P* = 0.0046; F(3, 14) = 6.806; DF = 3) (Figure 6B).

###  Difference Score Results

 As shown in Figure 6C, the hypoxia group treated with ExoC1-RBP-C spent significantly (*P* < 0.05) more time investigating the novel object and reduced time close to the familiar object in comparison to the control group, 24 hours after hypoxia. No significant differences were observed between the other experimental groups (*P* = 0.0383; F(3, 14) = 3.680; DF = 3).

 The difference score for the duration spent near the novel and familiar objects in the hypoxic group was less than in the other groups, as this group spent notably less time near the new object compared to the control group (*P* < 0.05). Moreover, the difference in the duration spent close to the new and old objects showed a significant increase in the hypoxia group one-week post-injection of ExoC1-RBP-H, in comparison to the hypoxic group (*P* < 0.05) (*P* = 0.0076; F(3, 14) = 5.983; DF = 3) (Figure 6D).

###  Motor Control and Balance Results

 The results showed that the average balance and duration on the rotarod in the hypoxia group significantly decreased compared to the control groups. The hypoxia group spent less time on the rotating bar and exhibited poorer balance compared to the control group (*P* < 0.05) (*P* = 0.0408; F(3, 13) = 3.677; DF = 3). The balance time on the rotarod, measured one week after treatment, indicated that the mean balance retention and time spent on the rotarod were significantly lower in the hypoxia group compared to the control groups (*P* < 0.01). However, the time to maintain balance one week after injection of ExoC1-RBP-H in the hypoxia group showed a significant increase compared to the baseline measurements (*P* < 0.05) (*P* = 0.0016; F(3, 14) = 8.804; DF = 3) (Figure 6E and 6F).

###  Wire Hanging Test Results

 Post-RBP injection assessments showed a significant reduction in mean balance time for both hypoxia (*P* < 0.01) and hypoxic rats treated with ExoC1-RBP-C compared to controls (*P* < 0.05) (*P* = 0.0012; F(3, 18) = 8.141; DF = 3). However, hypoxic rats receiving ExoC1-RBP-H exhibited improved balance time (*P* < 0.05). One week later, mean balance time in the hypoxia group decreased significantly compared to controls (*P* < 0.05), but the ExoC1-RBP-H group showed a significant increase in balance time compared to untreated hypoxic rats (*P* < 0.05) (*P* = 0.0147; F(3, 18) = 4.604; DF = 3) (Figure 6G and 6H).

## Discussion

 In addition to the UV-Vis spectral results, the prepared Fe-RNA conjugates exhibit characteristic changes in the amide groups of Fe and the phosphate groups of RNA. These changes are consistent with previous studies^30-33^ and indicate successful conjugation. Furthermore, the disappearance of carbonyl groups in RNA nucleotides after reaction with activated Fe-nanoparticles suggests another type of noncovalent interaction. This study investigated the use of exosomes derived from rat microglia as carriers of RNA-binding proteins (RBPs), their uptake by microglial cells, and their role as vehicles for transferring RBPs to the CNS. The results align with previous research indicating the uptake and effects of microglial exosomes on CNS cells.^13,14^ Additionally, the effects of exosomes containing RNA-binding proteins on microglial cell viability over a 14-day period, as well as their impact on the expression of genes related to cellular resistance to hypoxia and innate immune factors over 24 hours, were successfully evaluated. The pathological effects of hypoxia can manifest acutely within the first 14 days, subacutely between 3 and 11 weeks, and chronically over periods of 3 to 6 months or beyond 6 months.^34^ Given the significance and prevalence of hypoxia across various age groups, as well as the complications associated with post-hypoxia inflammation, this study focused on a 14-day period to assess the impact of hypoxia on microglial cell survival.

 Many cells contribute to the repair process following cerebral ischemia, and exosomes secreted by these cells play a crucial role in hypoxic conditions. In the context of cerebral ischemia, exosomes can mitigate neuronal damage and enhance the brain’s microenvironment by regulating inflammation, promoting axonal growth, mediating pyropathosis (the death of inflammatory and immune cells), and stimulating angiogenesis. Consequently, exosomes may serve as potential therapeutic agents for ischemia.^35^ Therefore, in this study, exosomes were utilized as carriers of RNA-binding proteins (RBPs).

 The exosome contains a variety of proteins, including cytoskeletal proteins, tubulin, actin, heat shock proteins 70 and 90, metabolic enzymes involved in glucose metabolism, flutellin-1, signaling proteins such as kinases and heterotrimeric G proteins, MHC molecules, clathrin, transport and fusion proteins such as annexin and Rab proteins, translation factors, and tetraspanning family proteins, including CD9, CD63, CD81, and CD82, as well as cellular proteins from which they are derived. Microglial exosomes also contain N- aminopeptidase (CD13) and monocarboxylate transporter 1. Aminopeptidase removes amino-terminal amino acids from polypeptides, which degrades enkephalin. This process affects the activity of opioid receptor ligands and the cAMP levels in neurons. Lactate transporters, along with glycolytic enzymes, provide energy sources for neurons.^36^ The size of exosomes isolated from healthy brain microglial cells fell within the expected range of 20-200 nm and was consistent with findings from other studies.^37^

 The primary responsibility for modulating post-translational changes lies with RBPs.^38^ Deficiencies in RBPs disrupt RNA metabolism and can lead to inflammatory brain diseases associated with microglial and astrocytic activity, such as amyotrophic lateral sclerosis.^39^ Consequently, this study investigates the role of RBP in the development of hypoxia resistance. A proteomics analysis of rat brain tissue identified 526 RNA-binding proteins, of which 431 had been previously recognized across 14 mass spectrometry projects. Some of the proteins involved in metabolism and membrane binding were also among those for which the role of RBPs was confirmed in that project. In the mentioned study, oligo-DT-conjugated to the magnetic blades were utilized for isolation of RBPs.^40^ Additionally, another study identified the role of the RBP known as intracellular T antigen (TIA1) in reducing brain inflammation and decreasing the activity of innate immune cells, which in turn diminishes apoptosis and cytotoxic inflammation in stressed cells.^41^ In conjunction with the aforementioned studies, the transfer of RBPs enhanced the activity of the recipient cells, showing no significant difference in viability compared to the control group. However, after 7 days, a transient decrease in the viability or metabolism of these cells was observed, which may be attributed to the half-life of the transferred proteins and their degradation within the recipient cells after the initial protective effects.

 Due to the negligible differences in viability among RBP-receiving cells across different groups during the study period, increasing the amount of RBP present in healthy and hypoxic brain recipient cells, or the effects of their vehicle exosomes, resulted in resistance to hypoxia. However, as discussed in the following sections, the expression profiles of protective proteins in the treated cells vary significantly among the different treatment groups. Therefore, when selecting the appropriate RBP to induce resistance to hypoxia, it is essential to consider not only viability assays but also the expression of inflammatory, anti-inflammatory, and hypoxia-resistance proteins. Two weeks after treatment, microglial cell viability did not differ significantly among RBP-receiving cells, hypoxia-exposed cells, and control cells. These results indicate the elimination of hypoxia effects in the treated cells and the recovery of their proliferative capacity over the two-week period. It is important to note that primary microglial cells proliferate in cell culture media but stop proliferating once they reach confluency, unlike malignant cells. This behavior may have influenced the results observed during the two-week evaluation period. The viable cells remaining after hypoxia induction proliferated and reached the same confluency as control cells during the two-week incubation. The significantly higher viability observed in the vehicle Exo-C1 group two weeks after treatment may be related to the metabolic status of these cells. Since Exo-C1 exhibited lower viability among all groups at the one-week evaluation, the surviving cells continued to proliferate until the two-week mark. In contrast, other groups reached optimal confluency earlier, resulting in lower metabolic activity compared to Exo-C1 at the two-week evaluation point.

 Entrance of the RBP-H to the hypoxic microglial cells significantly elevated the levels of the HIF-1α in these cells. In contrast, the other groups exhibited no significant changes or a decrease in HIF-1α expression. Therefore, transfer of RBP in the hypoxic microglial cell enhances the stability of HIF-1α RNA or its expression compared to the other groups. Increased expression of this gene has been documented in numerous studies following hypoxia induction. Other study indicates that HIF-1α protein levels rise immediately after hypoxic exposure, peaking at 3-4 hours post-hypoxic-ischemic injury, with elevated levels persisting for up to 24 hours thereafter.^42^ Kalesnykas et al. (2008),^43^ reported an increase in HIF-1α in rat neurons following unilateral occlusion of the common carotid artery. The reduction in blood flow and subsequent ischemia during this occlusion leads to cellular hypoxia, which stabilizes HIF-1α. In a study by Zhang et al. (2023),^44^ it was found that following transient hypoxia, microglial exosomes induce brain inflammation and cognitive impairment through miR-146a-5p-mediated activation of NLRP3 and HIF-1α. In the present study, control exosomes were utilized for RBP delivery. Notably, only RBP-H stabilized or increased the expression of HIF-1α, which corresponds to the requirements of the studied groups.

 The expression rate of HIF-1α varies under different hypoxic conditions. In a rat model subjected to hypoxia for 2.5 hours with 8% O2, Li et al. (2007),^45^ found that HIF-1α expression increased significantly following hypoxia. This suggests that hypoxic stimulation alone may induce greater HIF-1α expression than the combined stimulation of hypoxia and ischemia. This finding implies that HIF-1α plays a crucial role in maintaining brain integrity under hypoxic conditions. Also, in normal rat pups, HIF-1α expression increases between the 8th and 9th gestational days.^46^ The neonatal rats subjected to hypoxia and have neuron-specific deficiency of HIF-1α showed hydrocephalus, neuronal loss, and impaired spatial memory by 10 weeks of age.^47^ Consequently, the increase in HIF-1α in RBP-H treated cells may be regarded as a contributing factor to the development of resistance and a more effective response to hypoxia. Li et al. (2022),^48^ demonstrated that exosomes derived from human amniotic fluid significantly increased the expression of HIF-1α and VEGF in the cerebral cortex of rats. In vivo tests showed that oxygen and glucose deprivation created pores that facilitated the passage of HUVEC cells and improved hypoxic encephalopathy in newborn rats. In the current study, microglial cells treated with vehicle exosomes exhibited a decrease in the expression of HIF-1α and VEGF. The variation in the source of exosomes is one of the factors contributing to this discrepancy. Both effects, including the reduction of HIF-1α and VEGF, were modulated by RBP-H. These findings show that, although healthy microglial exosomes reduced some of the protective genes related to hypoxia, the RBPs from hypoxic newborn rats stabilized or induced the expression of these protective genes.

 Transport of RBP-H to microglial cells significantly increased the RNA levels of the CPE gene. CPEB, as an RBP with the ability to bind to specific RNA sequences, enhances polyadenylation and translation in neurons. Research on CPEB1 in microglia has shown that rats lacking this molecule exhibit increased IL-6 secretion, heightened inflammatory responses, and enhanced phagocytosis in response to lipopolysaccharide.^49^ Therefore, the increase in CPE gene expression following treatment with RBP-H can be considered a contributing factor to cellular stability and the reduction of inflammation. It is noteworthy that this increase was observed at a lower rate following treatment with RBP-C and RBP-C1. In addition, CPE plays multiple roles in the central nervous system, including the maintenance of normal cognitive function, proper neural structure, and neuronal survival.^50^ Consequently, rats lacking CPE activity exhibit various neurological and behavioral abnormalities. Also, the processing and sorting of neuropeptides in the cerebral cortex may be disrupted due to the aberrant accumulation of CPE.^46^ It appears that memory enhancement in the group of rats treated with RBP-H is associated with the induction or stability of CPE gene expression.

 Given the protective effects of HSPs, researchers have suggested that stimulating cells to enhance HSP expression may offer a strategy to shield the brain from degenerative diseases.^51^ HSP70 is also recognized as a valuable marker for cellular responses to hypoxia. Recent studies have explored the neurobehavioral functions of HSP70 during chronic hypoxiaand its protective role in cerebral ischemia.^52^ Furthermore, HSP70 is implicated in various abnormalities of the immune response, which is why it is considered in the context of many inflammatory diseases to help regulate inflammation. However, increased non-selective expression of HSP70 can lead to adverse effects by interfering with the functions of other proteins.^53^ In experiments where cells were treated with RBP, a decrease in HSP70 expression was observed compared to the untreated group. Considering the survival rates of the cells during the study period relative to the control group, the reduction of hypoxic stress in the treated cells may explain the decrease in the RNA levels of HSP70.

 The PDI gene is expressed in glial cells under hypoxic conditions and in cells affected by cerebral ischemia. The experimental studies involving the transfer of this protein to the brains of rats have demonstrated that increased levels of PDI during hypoxia and ischemia confer cellular resistance and prevent cell death.^54^ Additionally, PDI has been identified as a potential biomarker for diagnosing pathological conditions associated with Alzheimer’s disease in cerebrospinal fluid.^55^ Furthermore, mutations in the PDI gene have been linked to disorders affecting the development of the nervous system and memory.^56^ Inhibition of PDI function or disruption of PDI through RNA interference (iRNA) leads to the inhibition of NOx activation in microglial cells, resulting in decreased superoxide production and increased secretion of TNF-α. Elevated expression of PDI is associated with inflammatory brain diseases, likely due to its role in maintaining the integrity of oxidative proteins within the endoplasmic reticulum.^57^ This information provides a dual perspective on the role of PDI in neuroinflammation, encompassing both the prevention of cell death and the induction of inflammation. The current study demonstrated that the RBPs from both newborn rats aged 7 days induced PDI expression. However, six days later, the purified RBPs from these groups exhibited a reduced effect on PDI gene expression stability compared to earlier ages. Additionally, results showed decreased expression of this gene in cells treated with the H and H1 groups compared to healthy C and C1 treated cells. Therefore, the RBPs from the H and H1 groups, by reducing PDI levels, could alleviate inflammation during hypoxia; however, due to the hypoxic condition of the cells, the expression level remained higher than in control cells. In agreement, Tian et al. (2009),^58^ reported that exposed rats to chronic hypoxia exhibited increased expression of PDI in capillary endothelial cells located in healthy regions surrounding infarctions and myocardial infarctions. This finding underscores the significant role of PDI in the formation of new blood vessels. The aforementioned study also indicated that exposure to chronic hypoxia conferred protection against myocardial infarction. Zhang et al. (2021),^59^ the injection of serum exosomes from young rats aged 3 months into older rats with experimental cerebral ischemia aged 21-23 months resulted in improvements in both short-term and long-term complications. Conversely, the administration of serum exosomes from older rats with ischemia exacerbated complications due to increased phagocytosis by microglial cells. The deficiency in a physiological requirement for high expression of RBPs in older individuals, compared to the newborn stage, has led to a decrease in the RBP expression in the C1 group compared to C group.

 Cerebral ischemia stimulates the expression of vascular endothelial growth factor (VEGF) and its receptors. Marti et al. (2000), found that 48 to 72 hours after the permanent occlusion of the middle cerebral artery, the number of newly formed blood vessels at the border of the infarction increases significantly. VEGF expression is induced by hypoxia and is markedly elevated at the ischemic border between 6 and 24 hours following vascular occlusion. Two transcription factors, HIF-1 and HIF-2, which play a crucial role in regulating the expression of VEGF and VEGFR genes, show increased levels at the ischemic border after 72 hours, indicating a regulatory function for these factors. These findings suggest that the VEGF/VEGFR system, activated by hypoxia, contributes to the growth of new brain vessels.^60^ The stability of VEGF under hypoxic conditions is dependent on the levels of human antigen R (HuR). In this study, the highest levels of VEGF were observed in the hypoxia group receiving RBP-C1, while the lowest levels were found in the group treated with vehicle exosomes. Therefore, the increase in VEGF may be attributed to the influence of RBPs on RNA stability or to enhanced VEGF expression through the modulation of transcription factors. Elevated VEGF expression has been associated with behavioral abnormalities in autism and brain inflammation.^61^

 Circulating innate immune cells may respond to hypoxia through the activation of nuclear factor-κB (NF-κB), which initiates inflammatory responses. Neutrophil and macrophage responses include the secretion of degradative enzymes.^62^ Consistent with previous reports,^63-65^ the current study showed a non-significant increase in lysozyme and myeloperoxidase enzyme levels compared to normal newborn rats, with increases of 25.76% and 12.35%, respectively. The elevation of degradative enzymes, combined with a non-significant decrease in serum antiprotease activity (14.6%) in hypoxic animals, may contribute to tissue degeneration following hypoxia. The differentiation of microglia into an inflammatory state or interactions between serum protease inhibitors and secreted protease enzymes could underlie the reduction in serum antiprotease activity observed in hypoxic animals. Notably, exosome-mediated delivery of RBP-H significantly increased antiprotease activity and reduced lysozyme and myeloperoxidase activity in hypoxic microglial cells.

 The results of the current study indicate that the hypoxia affected animals which were treated with RBP, exhibited improvements in memory, cognition, balance, and motor coordination compared to the hypoxia group. Furthermore, there was no significant difference between this treatment group and the control group. Conversely, the difference between the hypoxia group treated with RBP and the control group was not significant, which aligns with previous findings regarding the evaluation of microglial cell viability following RBP administration. Based on these findings, it appears that the administration of exosomes containing RBP from brain cells affected by neonatal hypoxia positively influences memory, cognition, balance, and motor coordination in rats. Hypoxia impairs cognitive function and motor skills by causing brain oxidative stress, inflammation, and nerve damage. It disrupts synaptic plasticity and neurogenesis, and compromises cognitive capabilities. Research indicates that inflammation activates microglia, contributing to nerve damage and impaired behavioral responses following hypoxic-ischemic injury in the developing brain, underscoring the detrimental effects of hypoxia on brain function.^66^ Studies have demonstrated the critical role of RBPs in neuroprotection and synaptic plasticity, vital for cognitive and motor functions.^7^ The current study demonstrates that hypoxia significantly impairs memory and motor balance in rats compared to controls. Memory deficits often result from neonatal hypoxia during puberty, which is identified as a leading cause of mortality associated with neurological disorders.^67^ Exosomes containing RBP from hypoxia-affected brain cells can protect hypoxic rats by improving cognitive and motor functions.^68^ These hypoxia-derived proteins may enhance neuronal survival by modulating gene expression. Future research should focus on elucidating the molecular pathways through which RBPs function under hypoxic conditions.

## Conclusion

 In addition to the resistance of microglial cells and modulation of their inflammatory status, this study highlights the therapeutic potential of RBPs for cognitive impairments related to hypoxia. Cells treated with ExoC1 + RBP-H exhibited the most significant effect on RBP expression. Improvements in antiprotease activity, along with decreased lysozyme and myeloperoxidase activities, were observed in this group. Additionally, enhancements in cognitive and balance factors were noted following the treatment of cells with these proteins. Therefore, these proteins may be considered a promising option for further studies aimed at treating brain disorders resulting from hypoxia. Based on the information mentioned, to utilize RBPs in therapeutic applications, it is essential to characterize the RBPs present in hypoxic individuals and to elucidate the precise mechanisms and pathways through which they exert their effects.

## Competing Interests

 The authors declare that they have no conflict of interest.

## Data Availability Statement

 All analyzed/raw data are available on request from the corresponding author.

## Ethical Approval

 All procedures involving animals were conducted in accordance with animal protection ethics and the guidelines of the research ethics committee of Shahid Chamran University of Ahvaz, Iran, which approved the study with verification code SCU.REC.1402.019.

## Supplementary File

 The supplementary file includes the group nomination (Supplementary Figure 1), the process of RNA-binding protein extraction and in vitro assays (Supplementary Figure 2), and the in vivo evaluation (Supplementary Figure 3).
