# Supplementary material for: The RNA-Binding Proteins Promote Resistance of Microglial Cells to Hypoxia
Source: Adv Pharm Bull. 2025 Dec 23;16(1):91–109. doi: 10.34172/apb.025.46054 (PMC13408389; doi:10.34172/apb.025.46054)
Supplement: Supplementary file 1 — The supplementary file includes the group nomination (Supplementary Figure 1), the process of RNA-binding protein extraction and in vitro assays (Supplementary Figure 2), and the in vivo evaluation (Supplementary Figure 3). [file apb-16-91-s001.pdf]

Adv Pharm Bull 2026;16(1):91-S1
doi:10.34172/apb.025.46054

https://apb.tbzmed.ac.ir

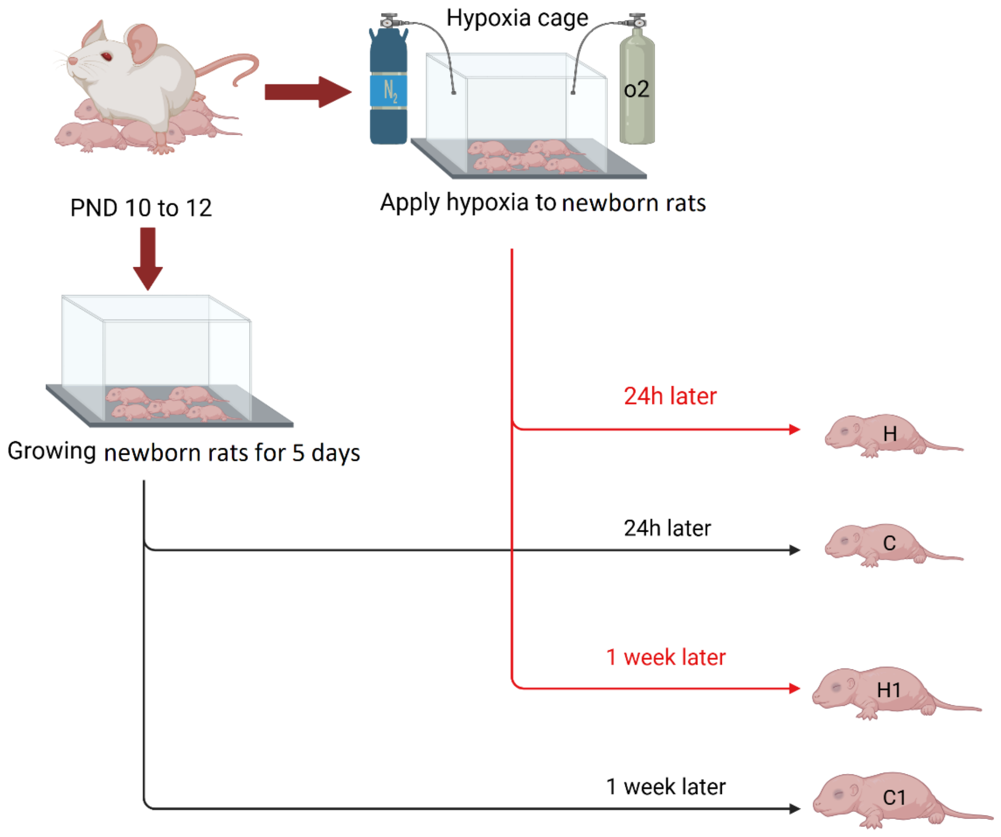

Supplementary Figure 1. Induction of hypoxia in newborn rats. The animals were divided into four groups, each consisting of five pups: hypoxia (H), one week after hypoxia induction (H1), control of hypoxia (C), and control one week after hypoxia induction (C1).

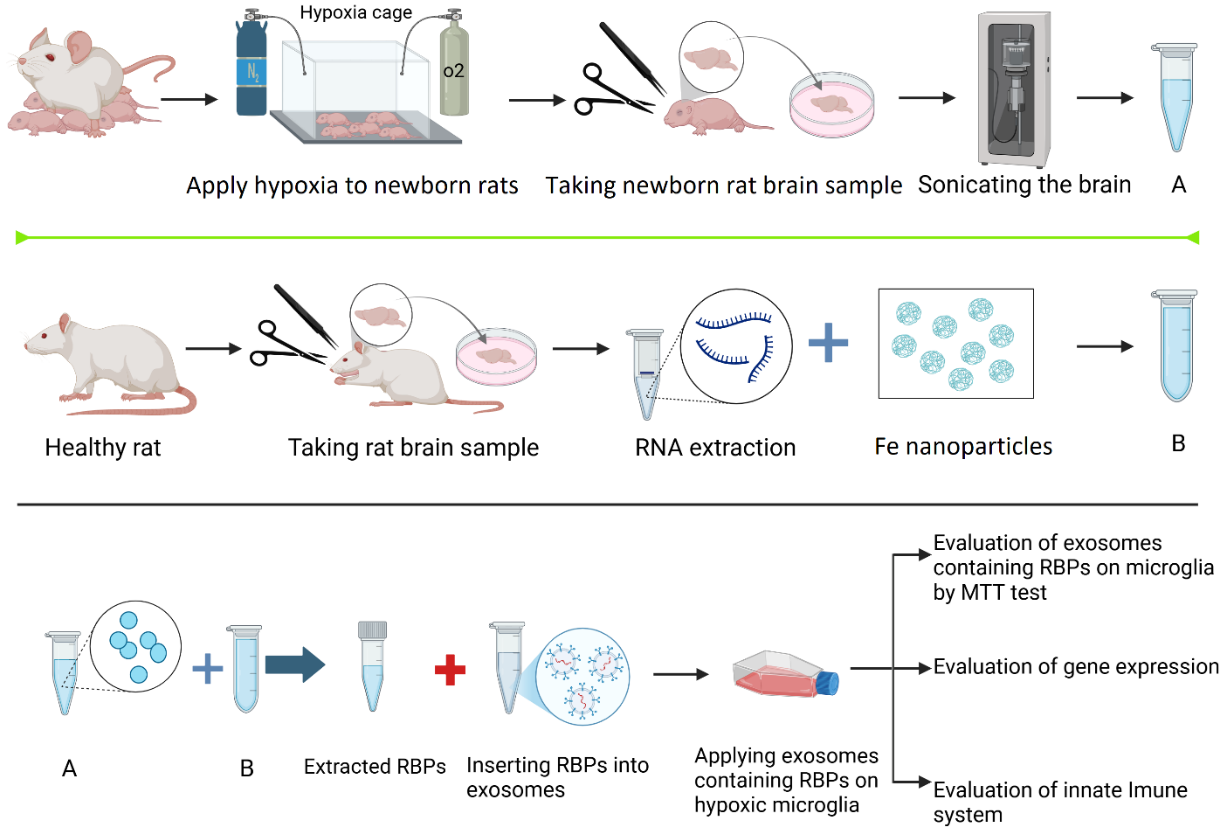

Supplementary Figure 2. Extraction of RNA-binding proteins from newborn rat brains subjected to hypoxia, utilizing Fe-nanoparticles conjugated to the RNA of the rat brains.

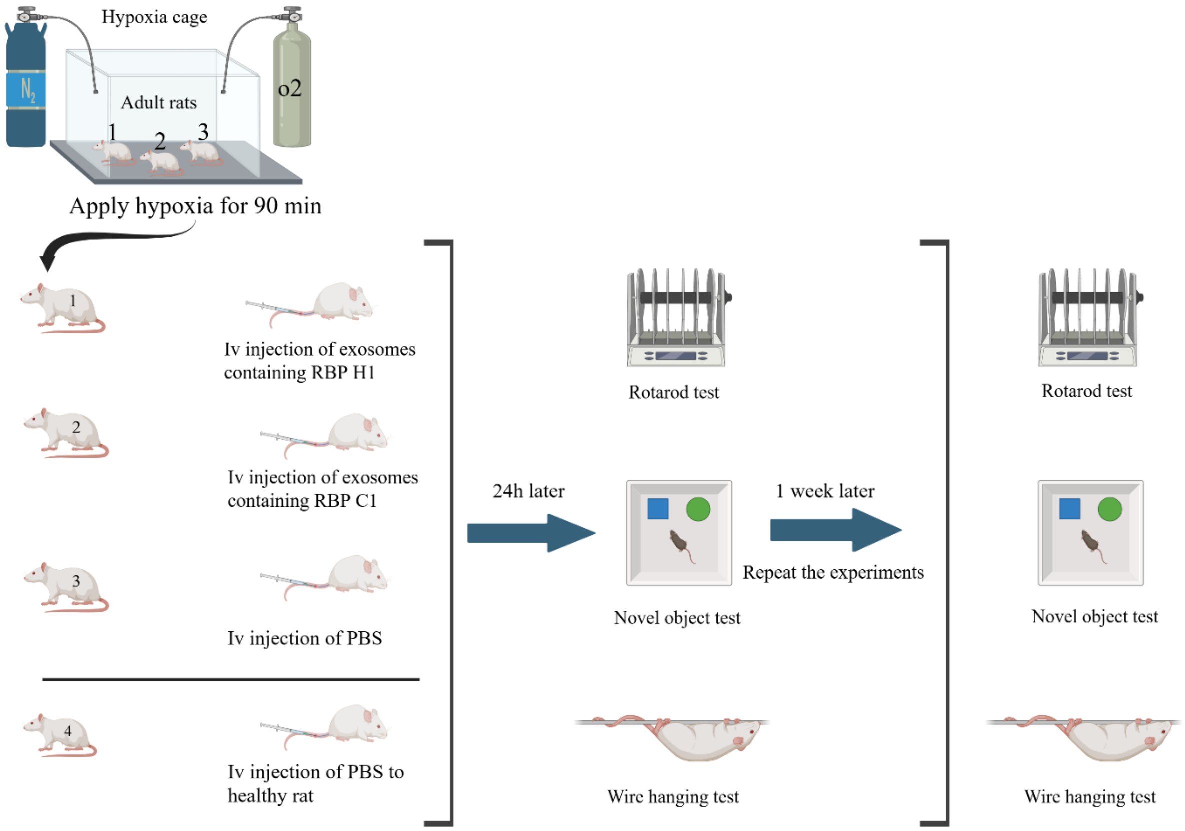

Supplementary Figure 3. Evaluation of the effects of microglial exosomes containing RNA-binding proteins on the behavioral characteristics of hypoxic rats, assessed in vivo using the rotarod test, novel object recognition test, and wire hanging test.
